# Plasma acetate, gluconate and interleukin-6 profiles during and after cardiopulmonary bypass: a comparison of Plasma-Lyte 148 with a bicarbonate-balanced solution

**DOI:** 10.1186/cc9966

**Published:** 2011-01-14

**Authors:** Paul G Davies, Balasubramanian Venkatesh, Thomas J Morgan, Jeffrey J Presneill, Peter S Kruger, Bronwyn J Thomas, Michael S Roberts, Julie Mundy

**Affiliations:** 1 Department of Anaesthesia, Princess Alexandra Hospital, University of Queensland, Ipswich Road, Woolloongabba, QLD 4102, Australia; 2 Intensive Care Unit, Princess Alexandra and Wesley Hospitals, University of Queensland, Ipswich Road, Woolloongabba, QLD 4102, Australia; 3 Intensive Care, Mater Misericordiae Hospital, University of Queensland, 550 Stanley Street, South Brisbane, Brisbane, QLD 4101, Australia; 4 Medicine and Pharmacology, University of Queensland, Chancellors Place, St Lucia, Brisbane QLD 4067, Australia; 5 Department of Cardiothoracic Surgery, Princess Alexandra Hospital, University of Queensland, Ipswich Road, Woolloongabba, QLD 4102, Australia

## Abstract

**Introduction:**

As even small concentrations of acetate in the plasma result in pro-inflammatory and cardiotoxic effects, it has been removed from renal replacement fluids. However, Plasma-Lyte 148 (Plasma-Lyte), an electrolyte replacement solution containing acetate plus gluconate is a common circuit prime for cardio-pulmonary bypass (CPB). No published data exist on the peak plasma acetate and gluconate concentrations resulting from the use of Plasma-Lyte 148 during CPB.

**Methods:**

Thirty adult patients were systematically allocated 1:1 to CPB prime with either bicarbonate-balanced fluid (24 mmol/L bicarbonate) or Plasma-Lyte 148. Arterial blood acetate, gluconate and interleukin-6 (IL-6) levels were measured immediately before CPB (T1), three minutes after CPB commencement (T2), immediately before CPB separation (T3), and four hours post separation (T4).

**Results:**

Acetate concentrations (normal 0.04 to 0.07 mmol/L) became markedly elevated at T2, where the Plasma-Lyte group (median 3.69, range (2.46 to 8.55)) exceeded the bicarbonate group (0.16 (0.02 to 3.49), *P *< 0.0005). At T3, levels had declined but the differential pattern remained apparent (Plasma-Lyte 0.35 (0.00 to 1.84) versus bicarbonate 0.17 (0.00 to 0.81)). Normal circulating acetate concentrations were not restored until T4. Similar gluconate concentration profiles and inter-group differences were seen, with a slower T3 decay. IL-6 increased across CPB, peaking at T4, with no clear difference between groups.

**Conclusions:**

Use of acetate containing prime solutions result in supraphysiological plasma concentrations of acetate. The use of acetate-free prime fluid in CPB significantly reduced but did not eliminate large acetate surges in cardiac surgical patients. Complete elimination of acetate surges would require the use of acetate free bolus fluids and cardioplegia solutions.

**Trial registration:**

Australia and New Zealand Clinical Trials Register (ANZCTR): ACTRN12610000267055

## Introduction

Open heart surgery utilizing cardio-pulmonary bypass (CPB) is the most common method of cardiac surgery worldwide [1]. A potential complication [2] of CPB is metabolic acidosis, which is influenced by the CPB circuit priming fluid [3,4]. Attempts to prevent metabolic acidosis have entailed alterations to circuit prime fluids, including partial replacement of chloride by rapidly metabolised anions such as L-lactate [5,6], acetate and gluconate [3,7], or else by bicarbonate [8]. Plasma-Lyte 148 (Baxter, Toongabbie, NSW, Australia) is a crystalloid CPB prime fluid in common use in Australia and New Zealand (personal communication, Andrew McLaren, Baxter, Australia) in about 7% of surveyed units in the UK [9] and in some units in North America [10]. One litre of Plasma-Lyte 148 contains 27 mmol acetate and 23 mmol gluconate. In a recent Phase II evaluation of Plasma-Lyte 148 versus a bicarbonate balanced prime, CPB commencing with Plasma-Lyte 148 prime was associated with an immediate surge in unmeasured anions of >10 mEq/L, with residual elevations still present just prior to CPB cessation [8]. Liskaser and colleagues have reported a similar phenomenon [3]. In both studies the unmeasured anions were considered to be acetate and gluconate; however, neither study measured individual concentrations of either anion at any time point.

Acetate surges in the vasculature may not be benign. For example, in renal replacement therapy, the pro-inflammatory, vasodilatory, myocardial depressant and hypoxaemia promoting properties of acetate [11-17] led to its removal from contemporary renal replacement fluids. Nevertheless, acetate remains an integral component of commonly used CPB pump prime solutions. Little is known of the acetate concentration profile and consequent physiological impact during cardiac surgery incorporating exposure to acetate-based fluid. The situation concerning gluconate is equally unclear, despite its widespread use.

In this context, we measured concentrations of acetate and gluconate before, during and after CPB primed with either Plasma-Lyte 148 or an alternative bicarbonate-balanced crystalloid preparation. We also collected preliminary data on plasma IL-6 concentrations as a marker of the systemic inflammatory response accompanying CPB.

## Materials and methods

The protocol was approved by the Human Research Ethics Committee of the Princess Alexandra Hospital and written informed consent was obtained from the patients prior to enrolment (Australian New Zealand Clinical Trials Registry number ACTRN12610000267055).

Patients scheduled for elective cardiac surgery were eligible for enrolment. Exclusion criteria were an abnormal pre-operative venous plasma bicarbonate concentration (<22 mmol/L or >27 mmol/L), hypercapneic respiratory failure, an elevated plasma creatinine concentration (>120 micromol/L), diabetes mellitus or chronic liver disease.

Patients were assigned systematically to Plasma-Lyte 148 or bicarbonate fluid according to the identity of the available whole-body perfusionist. In the absence of investigator PGD as perfusionist, patients received cardiopulmonary circuit prime as normal with 2 L of Plasma-Lyte 148. In the presence of investigator PD as perfusionist the CPB circuit was primed with 2 L of bicarbonate-balanced fluid made up in the pump immediately prior to priming the circuit by sequential addition of 0.9% saline 1,130 mL, 0.45% saline 822 mL (Baxter Healthcare, Toongabbie, NSW, Australia) and 8.4% sodium bicarbonate 48 mL (Astra Pharmaceuticals, Sydney, NSW, Australia). The electrolyte compositions of bicarbonate-balanced fluid and Plasma-Lyte 148, along with the cardioplegia solution employed, are set out in Table 1.

**Table 1:** Composition of cardiopulmonary bypass circuit priming fluids and cardioplegia solution

	Plasma-Lyte 148	Bicarbonate-balanced	Cardioplegia
Na^+^	140	140	140
Cl^-^	98	116	256
K^+^	5	0	83
Mg^2+^	3		80
HCO_3_^-^		24	
Acetate	27		28
Gluconate	23		24

All concentrations in mmol/L.

The CPB circuit incorporated a membrane oxygenator (Dideco Avante, Cellplex, Sorin, Mirondola, Italy) and heart lung machine (Jostra HL 20, Maquet Critical Care AB, Solna, Sweden). The standard pump rate was 2.4 L/minute/m^2 ^and the target temperature ranged from 32°C to 35°C. Arterial blood was sampled at four time points:

T1, immediately before commencing CPB; T2, two minutes after commencement of CPB, prior to placement of the aortic cross-clamp; T3, on rewarming, just prior to separation from CPB; T4, four hours after separation from CPB.

Measurements at each time point included plasma acetate, gluconate and IL-6 concentrations. Acid-base data were also collected, and are the subject of a separate report. No fluids apart from the patient's allocated prime (Plasma-Lyte 148 or bicarbonate-balanced) were infused in the interval between T1 and T2. Between T2 and T3, cardioplegia was administered. In accord with standard clinical practice at the research centre, an additional Plasma-Lyte 148 boluses were administered to any patient regardless of experimental group if volume supplementation was required.

### Acetate and gluconate assay

Plasma acetate and gluconate concentrations were measured by commercially available kits (Megazyme, Minto, NSW, Australia). Acetate determination is based on the formation of NADH which is measured by the increase in absorbance at 340 nm. Gluconate determination is based on the amount of NADPH formed in the reaction which is stoichiometric with the amount of D-gluconic acid and measured by the increase in absorbance at 340 nm.

### IL-6 assay

Plasma IL-6 was measured using an ELISA technique (Jomar Bioscience Ltd, Kensington, SA). The detection antibody was a biotin conjugate antihuman IL-6. Recombinant human IL-6 was used as a standard.

The laboratory scientists performing the acetate, gluconate and the IL-6 assays were blinded to the group allocation.

### Data analysis

Initial exploratory data analysis involved calculation of summary statistics (non-parametric (Wilcoxon), (*t*-test) and Fisher exact tests as appropriate) and construction of trajectory plots according to treatment group. Following inspection of these plots, group differences for the change from baseline at selected time points in continuous outcome variables were compared after adjustment for baseline (T1) values using a simple analysis of covariance (ANCOVA) regression model. Natural logarithmic transformations were applied to stabilize the variance of circulating acetate, gluconate and IL-6 levels.

Finally, a population-averaged generalized linear model using a generalized estimating equation (GEE) approach with an unstructured working correlation matrix and robust standard error estimates adjusted for clustering within individual subjects was applied to these longitudinal data to evaluate the overall associations of the vector of (log transformed) acetate, gluconate and IL-6 levels with treatment group across the four time points. Multiple imputation for missing log acetate data was used to support conclusions from the standard generalized linear model.

Statistical analyses were performed using SAS version 9.1 for Windows (SAS Institute, Carey, NC, USA) and Stata version 11.1 (StataCorp. 2010. Stata Statistical Software: Release 11.1. College Station, TX, USA: StataCorp LP).

## Results

The patients' demographic details, type of surgery, CPB and post-CPB data are summarized in Table 2 according to trial fluid treatment. The Plasma-Lyte 148 and bicarbonate-balanced treatment groups were similar with respect to age and sex, operative type, CPB times, clamp times, volumes of administered cardioplegia, EuroSCORE and Parsonnet indices, incidence of new post-operative atrial fibrillation and frequency of inotrope use. All patients were discharged from the cardiac post-operative unit within 24 hours, and all survived to hospital discharge.

**Table 2:** Patient characteristics according to study fluid

	Bicarbonate-balanced	Plasma-Lyte 148	
			
	*N *= 15	**N**_**P **_**= 15**	*P*
Age, mean (SD), *y*	68 (8)	68 (9)	1.0*
Sex, *male*	12	11	1.0^†^
Surgical type			
CABG	11	12	0.7^†^
Valve	4	3	1.0^†^
CPB time, mean (SD), *min*	81 (31)	80 (20)	0.93*
Clamp time, mean (SD), *min*	54 (29)	58 (20)	0.69*
Cardioplegia volume, mean (SD), *mL*	325 (137)	340 (121)	0.74*
Euro score, median [range]	3 (0 to 10)	3 (1 to 7)	0.9^‡^
Parsonnet score, median [range]	8 (3 to 22)	8 (3 to 26)	0.54^‡^
New onset atrial fibrillation, *n*	4	3	1.0^†^
Inotrope use, *n*	2	4	0.65^†^
ICU survival, *n*	15	15	1.0^†^
Hospital survival, *n*	15	15	1.0^†^

* T test.

† Fisher exact test.

‡ Wilcoxon rank sum test.

CABG, coronary artery bypass grafting.

### Plasma acetate and gluconate and IL-6

**Figure 1:**
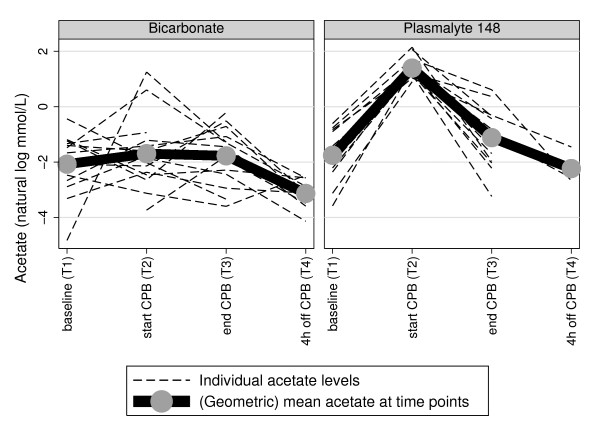
**Plasma acetate trajectory plots according to CPB prime, with geometric mean at each time point**.

**Figure 2:**
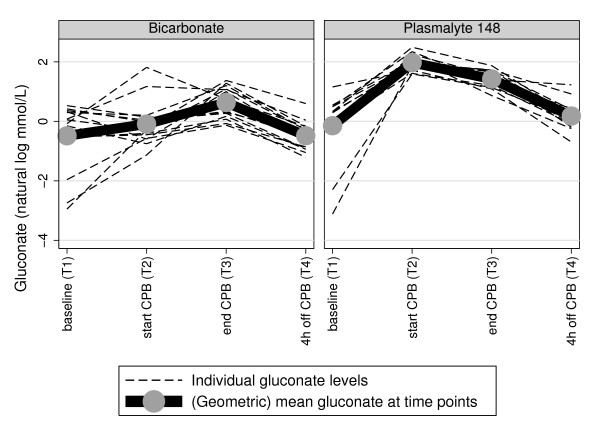
**Plasma gluconate trajectory plots according to CPB prime, with geometric mean at each time point**.

**Figure 3:**
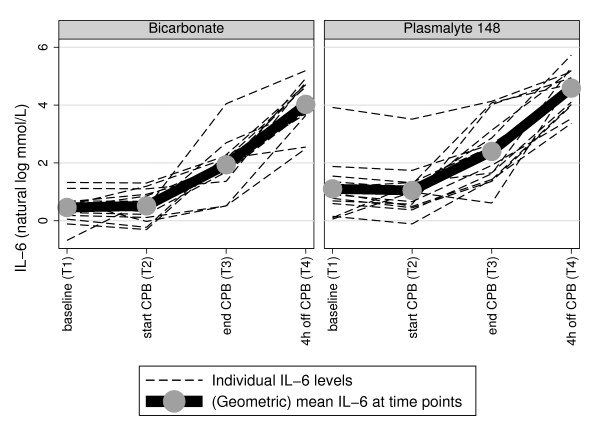
**Plasma IL-6 trajectory plots according to CPB prime, with geometric mean at each time point**.

Arterial blood acetate and gluconate and IL-6 levels, according to time of sample collection, are summarized in Table 3. As well, the time course of each individual subject's acetate and gluconate and IL-6 is illustrated respectively in Figures 1, 2 and 3

**Table 3:** Untransformed acetate, gluconate and interleukin 6 levels according to time of collection

Variable	Fluid		T1	T2	T3	T4
Acetate (mmol/L)	Bicarbonate	mean	0.17	0.49	0.24	0.03
		SD	0.17	0.94	0.23	0.03
		min	0	0.02	0	0
		median	0.13	0.16	0.17	0.03
		max	0.64	3.49	0.81	0.08

	P148	mean	0.21	4.29	0.49	0.03
		SD	0.18	1.76	0.53	0.06
		min	0	2.46	0	0
		median	0.18	3.69	0.35	0
		max	0.54	8.55	1.84	0.23

Gluconate (mmol/L)	Bicarbonate	mean	0.89	1.28	2.13	0.69
		SD	0.54	1.50	1.05	0.38
		min	0.05	0.32	0.88	0.30
		median	0.93	0.75	2.10	0.60
		max	1.70	6.13	3.95	1.82

	P148	mean	1.20	7.44	4.31	1.33
		SD	0.74	1.99	1.18	0.73
		min	0.04	4.92	2.38	0.50
		median	1.07	6.95	3.97	1.18
		max	3.18	12.1	6.51	3.42

IL-6 (pg/mL)	Bicarbonate	mean	1.75	1.88	10.3	72.5
		SD	0.81	0.92	13.4	49.7
		min	0.51	0.74	1.67	12.1
		median	1.69	1.55	7.14	47.9
		max	3.76	3.68	57.2	180

	P148	mean	5.99	4.72	19.2	119
		SD	12.4	8.07	21.4	74.5
		min	1.06	0.89	1.84	29.0
		median	2.65	2.7	12.5	113
		max	50.3	33.6	62.2	309

T1 Immediately before commencing CPB.

T2 Two minutes after commencement of CPB, prior to placement of the aortic cross-clamp.

T3 On rewarming, just prior to separation.

T4 4 hours after separation from CPB.

SD, standard deviation; Min, minimum value; max, maximum value; Bicarbonate, bicarbonate-balanced fluid; P148, Plasma-Lyte 148 fluid.

#### Acetate

Baseline (T1) arterial acetate concentrations were elevated above the normal range (0.04 to 0.07 mmol/L) in both groups [18]. These circulating acetate levels became markedly elevated at T2, where, accounting for the (log) acetate levels at baseline, there was very strong evidence (*P *< 0.0005) of a differential elevation of acetate at T2 compared to T1 in those allocated to Plasma-Lyte 148 compared to bicarbonate fluid. By T3, the substantially higher acetate concentrations in the Plasma-Lyte 148 group were diminished, but relatively normal concentrations were not restored in either group until T4 (Figure 1 and Table 3).

#### Gluconate

Overall gluconate levels were also greatest at T2 (Figure 2 and Table 3). Accounting for (log) gluconate levels at baseline, there was also very strong evidence (*P *< 0.0005) of a differentially elevated gluconate level at T2 compared to T1 for patients receiving Plasma-Lyte 148 relative to bicarbonate-balanced fluid. Subsequent circulating gluconate levels declined more slowly than for acetate in patients receiving Plasma-Lyte 148, to again approximate baseline values by T4 in both groups.

#### Interleukin-6

In contrast to acetate and gluconate, overall IL-6 levels increased progressively over time, reaching their maximum values at T4 (Figure 3 and Table 3). Although IL-6 levels were higher at T2 in the Plasma-Lyte group (*P *< 0.05), accounting for the (log) IL-6 levels at baseline, there was no strong evidence (*P *= 0.12) of a difference between the two groups in the change in circulating (log) IL-6 levels from T1 to T4.

### Overall population-averaged model

A marginal population-averaged linear regression model using the GEE approach also returned very strong evidence (*P *< 0.0005) of an independent differential association between administration of Plasma-Lyte 148 versus bicarbonate-balanced fluid as CPB prime fluid and elevated (log) circulating acetate levels across time, specifically at T2.

## Discussion

To our knowledge, this is the first report of serial plasma acetate and gluconate concentrations during and after CBP using Plasma-Lyte 148 prime. We found supra-physiological surges of both anions soon after institution of CBP with Plasma-Lyte 148, with persistent elevations at the end of bypass, particularly of gluconate.

This study documents previously suspected but incompletely characterized effects on serial plasma acetate and gluconate concentrations during and after CPB using Plasma-Lyte 148 as a CPB circuit priming fluid. It is noteworthy that in both groups, pre-bypass acetate concentrations were elevated at up to eight times previously reported normal human concentrations of 0.04 to 0.07 mmol/L [19]. Although increases of both anions were also observed in the group allocated to bicarbonate-balanced CPB prime fluid, these surges were smaller than with Plasma-Lyte 148. There are a number of potential explanations for these findings. First, several patients in both groups were likely to have received Plasma-Lyte 148 before the institution of CPB, as Plasma-Lyte 148 was at the clinical research site the default supplementary fluid for volume loading in either group. It is common practice at the Princess Alexandra Hospital to infuse approximately 1L of crystalloid at induction of anaesthesia. Second, all patients received on average over 300 mL of cardioplegia solution in early CPB prior to T3, with this solution containing acetate and gluconate in concentrations similar to Plasma-Lyte 148 (Table 1). Finally, elevated blood acetate concentrations have been reported in patients with type 2 diabetes mellitus [20]. Diabetes was an exclusion criterion for this study, but sub-clinical disease may have been present in some patients.

Although there are no comparative published human reference ranges, it is likely the pre-bypass circulating gluconate concentrations observed in this study were also above normal, related to pre-bypass fluid loading with Plasma-Lyte 148 in numerous patients. Gluconate is known to be eliminated more slowly [21] than acetate. The latter undergoes rapid metabolism at a rate of 300 mmol/h in health [22], with a significant extra-hepatic component [13]. This rapid clearance of acetate is preserved in experimental shock states [23], which may in part explain the choice of acetate in preference to L-lactate in 'balanced' resuscitation fluids such as Plasma-Lyte 148. Current data on gluconate metabolism and toxicity in humans are limited, and it is unknown to what extent CPB alters acetate or gluconate turnover.

Plasma IL-6 concentrations increased during and in the early hours following CPB in both groups. The greatest concentrations observed in this study were at four hours following cessation of CPB, consistent with previously reported maxima at three to six hours post CPB [24,25]. Unlike acetate and gluconate, IL-6 levels did not show strong evidence of differential changes over time between treatment groups, but the small size of the study precludes assessment of potentially subtle changes.

It remains unresolved whether the demonstrated supra-physiological concentrations of either anion could cause harm. The answer cannot be found in this small non-randomised, incompletely matched cohort study, particularly since substitution of bicarbonate in the circuit prime failed to eliminate exposure to supra-physiological concentrations of acetate and gluconate, for reasons already discussed. However, there is already unequivocal evidence of acetate toxicity in contexts other than CPB. A number of studies have documented hypoxia and hypotension when patients with end stage renal disease were dialysed against solutions containing acetate [12,13,15]. There is also evidence of cytokine release, carbohydrate intolerance, disturbances of fatty acid synthesis, reduction of cytosolic redox potential, intracellular accumulation of phosphate, pyrophosphate, phosphorylated intermediates and calcium, and deposition of intra-mitochondrial calcium and magnesium pyrophosphate [13].

Acetate has also been implicated in direct myocardial toxicity. Patients with chronic renal failure receiving acetate-free haemodiafiltration achieved better stroke volumes, demonstrated a lesser reduction in peripheral resistance and recorded smaller troponin increases than patients receiving conventional acetate-based dialysis [17]. In an isolated perfused rat heart model, exposure of myocardial tissue to acetate concentrations as low as 5 mmol/L resulted in impaired fatty acid oxidation and decreased ATP turnover [16]. Finally, Plasma-Lyte 148, although promoted as a resuscitation solution, performed poorly in a haemorrhagic shock model. Traverso *et al. *compared four resuscitation crystalloids [26], and found that Plasma-Lyte148 was associated with a lower survival and a late rise in plasma L-lactate concentrations as compared with normal saline and Ringer's lactate solutions.

Acetate-based haemodialysis in Australia and elsewhere has been abandoned, even in supplementary concentrations although it continues to be an integral component of 'balanced' resuscitation fluids, CPB priming solutions and total parenteral nutrition. During CPB, adverse effects triggered by acetate exposure would be difficult to detect amongst the vigorous metabolic and host defence responses to surgery [18,27], hypothermia, and non-pulsatile blood flow [1]. Although there is no proven detrimental effect, the concentrations documented in this report suggest a need for further investigations into the safety of acetate containing fluids during CPB. Even more importantly, these investigations should also address the practice of infusing high concentrations of acetate directly into the coronary circulation as part of cardioplegia solution.

Surges in circulating gluconate levels observed in this study were also marked, especially in the Plasma-Lyte 148 group. Unlike acetate, there is no current evidence of clinical or experimental gluconate toxicity, although information is limited. Animal data suggest that at concentrations of 2.4 and 4.8 mmol/L, gluconate may provide protection against post ischemic myocardial dysfunction and oxidative injury [28]. Gluconate has also been incorporated into some enteral and parenteral pharmaceutical preparations. The principal benefit of bicarbonate-balanced fluids demonstrated thus far, apart from avoidance of exogenous anions, is the ability to achieve a well balanced acid-base status across CPB [8]. Also, there is now evidence that added sodium bicarbonate may exert a renal protective effect in CPB [29]. The main difficulty associated with the use of bicarbonate is the need to prevent CO_2 _loss during preparation and storage [30], and the tendency for precipitation on exposure to small concentrations of calcium and magnesium.

## Conclusions

The use of Plasma-Lyte 148 as a prime fluid resulted in supra-physiological concentrations of acetate and gluconate across CPB. A bicarbonate-balanced pump prime fluid substantially reduced but did not eliminate surges of both anions, due to uncontrolled, clinically-directed administration of fluids containing acetate and gluconate, especially cardioplegia solution. While no clear differences in systemic inflammation were demonstrated, as reflected by circulating IL-6 levels, larger scale studies would be required for more precise assessment of this possibility. Currently, the use of acetate-free prime fluid in CPB cannot completely eliminate clinical exposure to large acetate surges until acetate is also removed from all other fluids used in CPB, including cardioplegia and bolus fluids. The impact of large plasma concentrations of gluconate also needs further elucidation.

## Key messages

• As even small concentrations of acetate in the plasma result in pro-inflammatory and cardiotoxic effects, it has been removed from renal replacement fluids.

• Acetate containing crystalloids are commonly used as prime solutions in cardiopulmonary bypass.

• This study has identified that supraphysiological concentrations of acetate and gluconate are reached in the plasma of patients undergoing cardiopulmonary bypass with Plasma-Lyte 148 as a prime solution.

• The use of acetate-free prime fluid in CPB significantly reduced but did not eliminate large acetate surges in cardiac surgical patients.

• Complete elimination of acetate surges would require the use of acetate free bolus fluids and cardioplegia solutions.

## Abbreviations

CABG: coronary artery bypass grafts; Cl^-^: chloride; CPB: cardiopulmonary bypass; GEE: generalized estimating equations; HCO_3_^-^: bicarbonate; IL-6: interleukin-6; K^=^: potassium; Na^+^: sodium; P148: plasmalyte 148.

## Competing interests

The authors declare that they have no competing interests.

## Authors' contributions

PD was involved in study design and study co-ordination, data analysis and manuscript preparations. BV was involved in overall study design and supervision, data analysis, and manuscript preparation and revision. JM was involved in study design, data analysis and manuscript revision. JP was involved in statistical analysis of data and manuscript revision. PK was involved in study design and manuscript preparation and revision. BT was involved in study design and sample collection. MR was involved in biochemical analyses, cytokine analysis and manuscript revision. JM was involved in study design, and manuscript revision. All authors read and approved the final manuscript.
